# A Framework for Future Analysis of Ophthalmology Fellowships in Iran: Call for Action, Implications and Recommendations

**DOI:** 10.18502/jovr.v19i1.15445

**Published:** 2024-03-14

**Authors:** Mohammad Ali Javadi, Shima Tabatabai

**Affiliations:** ^1^Ophthalmic Research Center, Research Institute for Ophthalmology and Vision Science, Shahid Beheshti University of Medical Sciences, Tehran, Iran

**Keywords:** Educational Planning, Fellowships, Future Trends, Health Priorities, Need Assessment, Ophthalmic Education

## Abstract

Ophthalmology fellowship is focusing on the educational advancement, medical research progress and academic productivity by transforming general ophthalmologists into superior clinical capacities in ophthalmology. There is a vast majority of ophthalmologists who wish to undertake fellowship degrees. The fellowship programs have several benefits for ophthalmologists and medical institutions. However, the expansion of ophthalmic fellowships has resulted in a greater number of ophthalmology visits, the possibility of unnecessary subspecialty eye examinations, induced demand and increasing eye-care costs. Moreover, sub-specialized ophthalmic services are not accessible to patients in remote regions. This can lead to a degree of inequity in the provision of healthcare services in the healthcare system. The massive expansion of fellowships in ophthalmology is revitalizing the necessity for evaluation of the need for post-residency education and providing effective planning for the future of the ophthalmic human-resource for eye health.

This narrative review includes an integration and descriptive summary of the existing evidence on trends and different aspects that affect the future of ophthalmic fellowship education. Moreover, we pinpointed challenges such as maintaining standards in fellowship education, keeping an efficient production of graduates, and improving productivity in both patient care and education. We explored potential solutions to overcome these challenges. The 7-step framework for future analysis suggested here includes Determining educational needs and desired outcomes, Evaluating the current status of fellowship education, determining the gaps, and appropriate solutions, analyzing possible future trends and their impact on ophthalmology practice, investing in virtual educational technology, developing new educational horizons by foresight expert panels, and human-resource planning.

##  INTRODUCTION

The Ministry of Health and Medical Education (MOHME) in Iran first offered post-residency medical training fellowships in the academic year 1984–85.^[1]^ One of the goals of these fellowship programs was to assist with faculty recruitment and academic development by focusing on the skills needed to have a successful career as a clinician educator, and to initiate serving as role models of excellence, professionalism, and scholarship.^[1,2]^

Fellowship educational programs in ophthalmology, like other post*-*residency fellowships, focus on the educational advancement, medical research progress, and academic productivity by transforming general ophthalmologists into superior ophthalmology clinical educators.^[2,3,4]^ There is a vast majority of ophthalmologists who wish to undertake fellowship degrees.^[4]^

The literature suggests that the advantage of formal post-residency fellowship education for both physicians individually and for medical universities are multifaceted.^[2,4,5,6,7]^ Ophthalmologists' gains include increased motivation for pursuing an academic career and professional development; improved mentorship skills through the use of advanced educational and assessment methods; and enhanced qualifications in becoming an independent educational scholar.^[3,4,5,6,7,9]^ Medical universities' advantages include training highly productive skilled researchers and educational leaders, improved educational tasks, extensive scientific publications, and community accountability.^[6,7,8]^

Post-residency medical education fellowships have recently become a topic of huge importance to international communities. Current literature also supports the importance of medical education fellowships and sub-specialized medical human-resources.^[2,3,4][5][6,7,8]^ However, post-residency medical education fellowships are heterogeneous when evaluating the educational needs.^[6,8,12]^

In Iran, there has been steady and sustained expansion of the fellowship education in the various ophthalmology subspecialty areas since the implementation of the programs.^[7,9]^ Standard criteria exist for accreditation of the post-residency fellowships including a curriculum for each fellowship discipline, an entrance examination, and admission process in Iran;^[1][7,9]^ while in some countries, there are no standard criteria for accreditation of post-residency fellowships as it pertains to program duration and curricular content.^[6,8,12]^

Although there is literature explaining why ophthalmologists undertake fellowship degrees and assessments highlighting the expectations of fellows regarding their training experience, there is no synthesis of the future needs of ophthalmic fellowship education.^[4,5,6,8]^ Nevertheless, most of the literature discussing the post residency fellowships in the 21st century are only concentrating on the current challenges.^[4,5]^ It is a crucial task of medical educators to see beyond the problems of today and to explore what may happen in the future.^[3,9]^ We as medical educators should evaluate the consequences of societal, technological, political, economic, or environmental changing trends that impact the delivery of sub-specialized eye-care, the practice, and their implications for assessing the requirements for ophthalmic fellowship education.^[9]^

A narrative review of all relevant papers known to the authors was conducted. Based on our review, we discussed the priorities needed to be addressed in the successful accreditation of post-residency ophthalmic education, and how they have applied to the determinants of the current and future fellowship curriculum. Furthermore, we considered the importance of educating competent and skillful ophthalmologists to meet the needs of the healthcare system in the future.^[11]^ Successful balanced expansion of the quantity of available ophthalmologists which requires substantial investment and continued research is a prerequisite for enhancing the ophthalmic care services system and improving public health outcomes.^[9,11]^

To give the readers an idea of the state of human resource planning in Iran's healthcare system with specific reference to ophthalmology, selected recent studies are briefly discussed below. Previous studies indicated that there was not one national planning forum to estimate the projected national need for more enrollment of ophthalmologists into the post residency fellowships or any mechanisms to define this need for different geographic regions in Iran.^[7,9][10]^ Furthermore, Iran's Health Evolution Program revealed that improvement of healthcare quality was out of reach due to the lack of sustainable resources, lack of adequate availability of health care professionals, and lack of necessary infrastructure, particularly in remote areas.^[11]^

In 2017, a national project entitled Iran Health Roadmap (NEDA 2026) was implemented using the record of the amalgamation of the experiences and predictions of experts in the healthcare field. One of the goals of the NEDA 2026 project was to create fair access to healthcare services in all fields including ophthalmology in Iran. This roadmap is the most important document that the Ministry of Health has adopted as its 10-year plan for healthcare in Iran. Some of the main components of this project were focused on clinical human resources and specialist personnel.^[11]^

Furthermore, our review did not discover any evidence of a prior study investigating either the future implications of experiencing expansion of fellowships in ophthalmology or determining areas needed for improvement in the fellowship education infrastructure in Iran.^[7,9]^ Our review discussed the importance of analyzing future implications of expansion of fellowship education. In addition, we proposed a framework to determine the implications of current trends on ophthalmic fellowship education, and we provided recommendations on how we can overcome any potential challenges.

##  METHODS

This narrative review and analysis includes an integration and descriptive summary of the existing evidence on trends and criteria of different aspects affecting the future of the fellowship programs in ophthalmology fellowship education. This study conducts an in-depth literature review of medical education literature published after 2002 using the optimal combination of electronic databases including Pub Med/ MEDLINE, Web of science core collection, Cochrane Library, and Google scholar.^[10]^ The following search terms were utilized: “ophthalmology” and “specialty”, “fellowship” or “medical fellowship” or “post-residency education” and “future”, “foresight”, “ future visioning”, “need analysis”, “trends”, “health challenges”, “trends”, “implications”, “sub-specialization”, “human-resources”, or “workforce” or “ophthalmologists”, “educational needs” or “training needs”, or “educational priorities.” A search for literature was also conducted in Iranian Farsi sources including Iran Medex and SID. The official reports of the MOHME were also accessed for information.

##  RESULTS AND DISCUSSION

**The Importance of Analysis of the Future Implications on Expansion of Fellowship Education in Ophthalmology**

It is important to analyze the needs and major purpose of the expansion of fellowship education in ophthalmology. A need analysis is executed to determine whether any root problems exist that may hinder the successful expansion of fellowship education in ophthalmology. Suggested recommendations arising out of the analysis are dependent on the feedback attained from the research. For instance, if the determined root problems stem from any educational gaps that exist between ophthalmologists' current qualifications and their training requirements that may affect their performance, the need analysts will recommend implementation of subspecialty ophthalmic curriculum as the solution.^[10,14]^ However, if the candidates possess a deficit in their knowledge and skills requirements, the increase in enrolment in ophthalmology post-residency fellowships will not assure effective performance or productivity.^[12,13,14,15,16,17]^

A comprehensive future analysis will also determine whether the operation of the ophthalmology departments impact on the productivity and performance of ophthalmologists including the presence of ineffective policies, low standards, unsecured medical devices, etc., which are not directly related to fellowship education.^[3,10,12,13,16,17]^ In this article, we propose a framework for the future analysis of fellowship education in ophthalmology which could reveal the educational and non-educational problems that need to be addressed.

***This framework suggests upper decision makers to address the following essential questions:***

How can fellowship education help assure access to healthcare in the future?

Why do ophthalmology departments continue to increase post-residency fellowship enrolment?

What do medical education policies call “efficient production of physicians”?

Is there, indeed, a saturation point for fellowship education in ophthalmology?

How the present expansion of ophthalmology fellowship education can best be managed?

How can the healthcare system assure the effective use of available human-resources in ophthalmology?

**The following 7 steps are suggested for an effective analysis of the future of fellowship education in ophthalmology:**

**Determining educational needs, priorities, and the desired outcomes in ophthalmology fellowships**

This step considers the acceptable performance of the ophthalmology subspecialist and the desired outcomes for fellowship education. Despite knowing the objectives that should be addressed in the ophthalmic fellowship curriculum, there is still the debate of which subjects of fellowship education are needed and which are really essential at this point.^[9,10,11,12,13]^

When conducting a needs analysis the following questions should be asked:

Which eye-care goals can be attained through post-residency ophthalmic fellowship education?

What will post-residency ophthalmic fellowship education cover?

What must the ophthalmic fellows learn in order to perform effectively?

Which ophthalmologists need fellowship training and for what?

In an effort to reach to accurate decisions, information was sought from trustworthy sources, such as the academic supervisors and clinical managers in ophthalmology departments, documentation related to educational tasks and practice, ophthalmologists' performance analysis and educational program evaluations.^[12,13]^

**Needs and priority themes for post-residency ophthalmic fellowship education programs:**

The competency-based education

The performance-based assessment

The use of virtual technologies

The evidence-based practices

Producing and disseminating research in ophthalmology

Promoting professionalism in ophthalmology education**

Promoting excellence in ophthalmic education for patient-centered care

**Determining the current outcome of fellowship education in Iran:**

In this step, we identify the current outcome of ophthalmic fellowship education and the actual performance of ophthalmologists with fellowship degrees (ophthalmic subspecialist human-resources**)** to determine whether the primary goals of the current programs are achieved.This step can be performed by reviewing the national reports and performance information documents, as well as by conducting interviews.

A national study revealed that post-residency fellowship programs are very attractive to general ophthalmologists and there is an increasing demand by specialists who wish to receive fellowship degrees in Iran.^[9]^ Most fellowships being offered are one to one and a half years programs that integrate both clinical and research experience.^[1,9]^

Fellowships related to ophthalmology are currently offered by eight medical sciences universities in seven fields of study in Iran, including: cornea and lens, vitreous and retina, glaucoma, pediatric ophthalmology and strabismus, oculoplastic, ocular pathology, and ocular surface disease.

After completion of fellowship training, the ophthalmologists often do not limit their practice to that sub-specialty area; rather they choose to integrate that area of expertise into their practice of general ophthalmology.^[9]^

**3. Determining the probable challenges, performance gaps, and the appropriate solutions: **In the process of executing the future needs analysis we should be proactive in approaching potential issues before they become actual problems. Once the challenge is identified, an argument turns up, to analyze the challenges and to reach a conclusion on what the system should do next.^[7][11][12,13]^ This step involves determining the expansion challenges associated with increasing demand for ophthalmic fellowship programs that can affect the current operation of the ophthalmic practices and by consequence, patient care, depending on ophthalmologists' motivation toward earning fellowship degrees. Identifying the challenges and the root causes facilitate evaluating an appropriate solution.

***Ophthalmology fellowship education challenges***

Maintaining standards in ophthalmic fellowship education, sustaining efficient production of fellows, improving productivity in both patient care and education, and minimizing the induced demand for services are some of the challenges associated with expansion of fellowships in ophthalmology education.^[9,12]^

Although ophthalmology fellowship programs aim to promote excellence in ophthalmic care for the whole population, ophthalmologists with fellowship degrees may only serve some specific patients with sub-specialized ophthalmic care demands.^[3][7,9]^ These advanced treatments would require the use of advanced medical equipment.^[9,13]^ It has also become clear that some patients do not have equitable access to sub-specialized medical services, including ophthalmic care, especially those patients who reside in remote communities and underserved regions. This would produce a degree of inequity in the healthcare system, in which patients who reside in remote regions and cities that possess less resources may receive less ophthalmic care and treatment than others fortunate enough to have access.^[15,16]^

Our review revealed that one of the most serious challenges in providing equitable service is facilitating access to advanced eye care. Access to healthcare has been recognized as the ability to visit a physician or to be hospitalized when in need of receiving care for prevention, treatment, amelioration, or palliation.^[17]^ Elderly persons constitute the largest age group of patients who require the services of ophthalmologists.^[18]^ However, a study revealed that almost 30% of elderly persons never saw an eye care provider within a five-year period.^[19]^ The majority of the patients, including candidates who were diagnosed with treatable conditions that may lead to potential vision loss have received eye care at least once in a five-year period, but did not access regular eye-care services.^[19]^

In order to address these challenges, the ophthalmology profession will need to consider redirecting the methods in which eye care is provided.^[17]^ Recommended solutions include promoting awareness of eye diseases and offering access to affordable quality medical and surgical eye care, in addition facilitating communications among ophthalmologists, eye care providers and elderly patients to enhance the provision and use of successful models of eye care around the country.^[15,16][17][18]^

Consequently, the current expansion of fellowships in ophthalmology have resulted in a greater number of patient visits in the more resourceful regions, increased possibility of executing unnecessary subspecialty eye examinations, in addition to increased eye care costs as a result of increased demand for ophthalmologists.^[15,16][17][18]^ These side effects are revitalizing the necessity for establishing need assessments for expansion of fellowship educational programs to assist in receiving maximum benefit.^[9][12,13]^

**4. Educational needs analysis by determining the trends which are changing the future of ophthalmology practice**

As the nature of ophthalmic care expands and evolves, so does the associated educational and infrastructural needs, which ensure successful service.^[12,13][14]^ Therefore, to develop an effective ophthalmology fellowship program, the ophthalmology departments should be prepared to transform their strategies toward informed development based on needs analysis which could identify gaps and offer solutions for “post-residency educational requirements in ophthalmology”.^[13,20,21,22,23]^

Required revisions in ophthalmic care needs and the working patterns of ophthalmologists are influenced by cultural and demographic transition, economic changes, evolving technology, changing work force, and the changing nature of ophthalmology science itself.^[7,9][14][21]^

Going forward, the practice of ophthalmology faces changing trends. Ophthalmology educational departments and decision makers need to recognize the possibility of future trends affecting the ophthalmology practice and prepare for appropriate educational developments.^[20,21,22,23,24,25]^

***As a result of our narrative review, the following factors were identified as affecting the future of the practice:***

(1) Demographic changes, (2) Epidemiologic transition, (3) Progress of technology, (4) Increased information, (5) Changes in patients' expectations of services, (6) Development of a different delivery model, (7) Innovation driven by competition, (8) Increase of eye care costs, (9) Changes in collaboration, communication, and practice guidelines, and (10) Continued need for a new care system.^[4,7,14,16,21,24]^

***The specific factors identified as influencing the advancement of ophthalmology fellowship education programs are:***

Ophthalmologists' generational transition;

Revisions to future ophthalmologists' personal values, lifestyle, and work patterns;

Multiple levels and variations in professionalism among subgroups of ophthalmologists/ophthalmologists in fellowship training;

Growth in the number of female ophthalmologists;

Changes in ophthalmologists' expectations of educational programs;

Growth in information and communication technologies; and

New advances in diagnostic and therapeutic technologies in ophthalmology.

***The consequence of current trends and its implications for fellowship education in ophthalmology***

Since the perspective of ophthalmic fellowship education is transforming, it is necessary to engage in futuristic plans to adapt to the impact of current trends affecting the future of post-residency ophthalmic education.^[9,21,24]^

***Ageing population*** **:**** As mentioned before, one of the most significant factors affecting future of ophthalmic care is **“Ageing population”.** The consequences for ophthalmology practice with respect to ageing populations include the possibility of increases in the prevalence and complexity of chronic eye diseases and disability. The possible implications for ophthalmology fellowship education include the need to train ophthalmologists who are prepared to pursue less attractive career paths: for example, primary eye care, geriatric eye care, and to work in partnership with patients in chronic disease management and self-care.^[22,23,24,25]^

The ophthalmology practice and training needs analysis provides reliable information for academic leaders to conclude on how to plan for the future of the ophthalmic fellowship programs.^[9,20]^ In addition, training needs analyses for post-residency ophthalmic education direct the ophthalmologists toward the right fellowship program by identifying the individuals who need further training and what training programs are appropriate to address their knowledge or skills gaps.^[9,23,24]^

***Generational differences*** **: **The future human-resource complement of the ophthalmology work force will include specialists and subspecialists from different generations. Ophthalmologists may also practice in dissimilar environments where they are required to adjust to cultural differences.^[21,26]^ These generational differences would impress upon ophthalmologists to exercise their understanding of professionalism, professional values, lifestyle and work patterns where the educational system would need to accommodate these transitions.^[20]^

***Professionalism*** **:**** The core competencies of professionalism among ophthalmologists such as the ophthalmologist–patient relationship, compassion, integrity, respect for others and social responsibility are learned through didactics, observation during education, and practical experience.^[26]^ A longitudinal study suggested that ophthalmologists' core competencies of professionalism enhanced with time through training and years of professional experience.^[27]^ The high level of professionalism among ophthalmology professors (who are role models for the trainees) will positively affect the level of professionalism expressed by the residents and fellows enrolled in ophthalmology educational programs.^[26,27]^

**5. Investing in virtual technology for competency-based education**

The transformative drivers embedded in virtual technology options are associated with meeting educational needs. They are likely to interact with other factors such as types of available educational resources and emergent medical/surgical technologies.^[3,9]^

Innovative learning technologies will change the future of ophthalmology education and new innovative strategies are needed when utilizing the acquired knowledge base to support competency-based education and assessment.^[3,9]^ Virtual reality (VR) technology provides practical support in competency based education and assessment (clinical/ surgical skills) in ophthalmology where it can respond to specific issues.^[28]^

Virtual simulation-based education products facilitate the necessary tools to provide benchmarking and best practice insights to ophthalmic fellows and residents, better preparing them for real-world practice.

One of the affordable platforms to improve knowledge and skill through simulation is mobile learning including the use of handheld and wearable devices. In addition, smartphone apps provide interactive learning resources for ophthalmology education.^[29]^

Furthermore, VR simulation could help integrate the ophthalmic fellowship education and assessment platforms.^[8,9]^ Medical sciences universities need to invest in individualized learning options for competency-based education and in the technologies necessary for virtual assessments of clinical competency. The advancements in VR technology as a training and assessment tool facilitates the execution of objective structured clinical examinations (OSCEs) and Virtual OSCEs (VOSCEs). VR simulation-based technologies contribute toward decreasing the cost of training while increasing the objectivity of the assessment processes.^[28]^

**Recommendations: What the system should do next****

In considering the impact of current and future trends, and their likely consequences and implications on post-residency fellowship education in ophthalmology, it is imperative that certain measures be implemented in order to plan for successful transitions in ophthalmic fellowship education programs and the associated human-resources element.

We recommend the following steps:

**6. Developing new horizons for ophthalmology fellowship education in Iran by Foresight Expert Panel (FEP)**

In this step, we should identify a plan for future direction of ophthalmic fellowship education in Iran. Future studies (foresight) attempt to examine not only what is possible, but also what is probable, establish preferred future goals and seek to gain a holistic or systematic view based on insights derived from multiple factors.^[9,20][21][22][33]^

Conducting a national foresight study with participation of top academic leaders and experts in ophthalmology education is suggested as health and medical education systems are often participatory. The assessment of predictable trends, however, will also have major implications for the future of post residency fellowship education.^[10,14]^

We suggest conducting a participatory foresight assessment with a qualitative approach by engaging the experts in ophthalmology education in long-term critical thinking to focus on trends that may form a probable future.

During the suggested foresight study with a recommended qualitative exploratory approach, the expert panel will discuss the challenges and outcomes of expansion of post-residency fellowship enrolment in Iran for the ophthalmology educational departments. Considering experts' viewpoints the significant challenges will be clarified. Then with an idealistic approach, the “horizons” for fellowship education in different ophthalmology fields over the upcoming decade will be determined.^[9,30,31,32]^ Therefore, national strategic foresight is necessary to ensure the effectiveness of fellowship education initiatives offered in different ophthalmology fields which will also encourage a significant shift toward purposeful development of the ophthalmology fellowship education curriculum in Iran.^[33]^

**7. Sharing discussions to shape the future ophthalmic fellowship human resources element**

This step should include discussion on the interplay between the current ophthalmology fellowship human-resources element and future ophthalmic service requirements, in the context of what is needed to secure high-quality and highly productive ophthalmic care for patients.^[9,18,30]^

These discussions require bringing in a broad scope of viewpoints on trends that will affect the nation's ophthalmologists, such as the socio-cultural, technological, environmental, economic, and political trends that can benefit the function of the ophthalmology fellowship human-resources planners who reflect through the implications.^[11,14,22]^

Addressing this challenge needs the involvement of all ophthalmology departments. Using methods to develop foresight will help ophthalmologists further enhance the productivity in their work. The expert recommendations will address community concerns about geographical inequity in ophthalmic service delivery.

The process of using foresight (future study) to determine the prerequisites of supply and demand for ophthalmic subspecialists assists in determining the scope for evidence collection, and information analysis that will enhance future research, to support and adjust the human-resources element in ophthalmology and addressing the eye care needs of the population.^[9,24,30]^ Future ophthalmologists will be better able to meet the needs of the population with access to this additional information.^[7,17,24,33]^

In this step, expert panels will determine the targets for each ophthalmic fellowship area considering the specific needs of each region's population and assessing the available ophthalmology facilities, services, and ophthalmic equipment.

***The expert panel will focus on several topics and will suggest several recommendations to***

Assure that we are using our ophthalmologist human-resources effectively.

Assure equitable access to ophthalmic care in the future.

Improve efficiency and effectiveness of expansion of fellowship education programs through evidence-based human-resources planning.

Improve the responsiveness of the ophthalmic subspecialty service to community health needs especially in underserved areas.

Improve the responsiveness of the ophthalmic services delivery to the needs of the ophthalmologists who are working in remote areas and also with specific reference to female ophthalmologists, offer flexible scheduling and part-time work.

##  SUMMARY

In this paper, we discussed the respective current and future trends and their probable implication on the infrastructure of ophthalmology education. We proposed a framework and recommendations to analyze training needs for post-residency fellowship education in ophthalmology and to plan for the future of ophthalmic fellowship education. Moreover, we explained the necessity of using the proposed framework for the adoption of foresight study in ophthalmology fellowship development policies, which will recommend solutions to the noted problems and unanswered questions.

Considering the next decade, ophthalmology education will continue to undergo significant change in structure. The progress of ophthalmic care will have important implications for ophthalmology departments not only in the practice and patient care mission, but also throughout all their educational and academic missions. This means, in order to assure high-quality ophthalmic education at specialty and sub-specialty levels, the academic administrators need to have foresight and plan for future ophthalmic care needs.

Moreover, education methods are evolving and new domains of ophthalmology sciences and technological innovations, as well as educational technologies, are expanding.^[22]^ Although, fellowship programs have the potential to ensure that transformative developments continue to benefit ophthalmic education, we will need to consider the emerging trends that impact the future approach that the ophthalmology departments will provide for the continued advancement of fellowship education.^[14]^

In conclusion, bringing in a broad scope of viewpoints on future needs to every discussion about the post-residency education programs is essential for medical educators to keep looking forward toward reforming the policies for promoting ophthalmic fellowship education and human-resource plans.

##  Financial Support and Sponsorship 

This study was funded by the Academy of Medical Sciences of I.R.IRAN.

##  Conflicts of Interest

None.
